# Pyogenic Spondylitis with Epidural Abscess Caused by *Streptococcus suis* Serotype 2 ST7: Tissue mNGS Confirmation and Whole-Genome Characterization of a Human Isolate

**DOI:** 10.3390/pathogens15030314

**Published:** 2026-03-13

**Authors:** Peiyan He, Henghui Wang, Ping Li, Yong Yan, Lei Gao, Lu Chen

**Affiliations:** 1 Jiaxing Key Laboratory of Pathogenic Microbiology, Jiaxing Center for Disease Control and Prevention, Jiaxing 314050, China; hepeiyan@aliyun.com (P.H.); wz345@sina.com (H.W.); ahliliping@163.com (P.L.); 13567309672@139.com (Y.Y.); shuangyuzuo007@126.com (L.G.); 2 Laboratory Department, Nanhu District Center for Disease Control and Prevention, Jiaxing 314050, China

**Keywords:** *Streptococcus suis*, zoonosis, serotype 2, sequence type 7 (ST7), pyogenic spondylitis, epidural abscess, metagenomic next-generation sequencing (mNGS), whole-genome sequencing (WGS)

## Abstract

*Streptococcus suis* is an emerging zoonotic pathogen that typically causes bacteremia or meningitis in humans, whereas vertebral osteomyelitis with epidural abscess is exceedingly rare and may be missed. We describe a 65-year-old farmer with fever and severe low back pain after long-term bare-handed handling of raw pig lungs. Pre-treatment blood cultures yielded *S. suis* identified by matrix-assisted laser desorption/ionization time-of-flight mass spectrometry (MALDI-TOF MS). After transient improvement on empirical therapy, fever recurred with worsening lumbar pain. Contrast-enhanced magnetic resonance imaging (MRI) demonstrated multilevel thoracolumbar pyogenic spondylitis with an epidural abscess and a sub-ligamentous abscess beneath the posterior longitudinal ligament (PLL) extending from L2 to L5. Computed tomography-guided lumbar biopsy followed by tissue metagenomic next-generation sequencing (mNGS) detected *S. suis*, providing concordant evidence supporting pathogen involvement at the vertebral focus. The bloodstream isolate (SS-JX2025-01) was serotype 2, sequence type 7 (ST7). It remained susceptible to β-lactams and glycopeptides but was resistant to macrolide–lincosamide and tetracycline classes, consistent with *erm(B)*, *tet(O)*, *tet(40)*, and *ant(6)-Ia* detected by whole-genome sequencing (WGS). Virulence profiling revealed an *epf*^+^/*sly*^+^/*mrp*^−^ pattern with multiple adhesins and immune-evasion factors, whereas canonical 89K pathogenicity island markers were absent. Core-genome phylogeny placed SS-JX2025-01 within the Chinese ST7 lineage associated with previous outbreaks. This biopsy-supported case expands the clinical spectrum of invasive *S. suis* infection, highlights the value of tissue mNGS as an adjunct for supporting deep-seated foci in zoonotic infections, and underscores the importance of occupational prevention in small-scale farming households.

## 1. Introduction

*Streptococcus suis* is a Gram-positive, facultatively anaerobic bacterium that colonizes the upper respiratory tract of pigs and causes invasive zoonotic infections in humans with occupational or dietary exposure [1,2]. Most human cases present with bacteremia or meningitis, frequently accompanied by sensorineural hearing loss; endocarditis, arthritis, and other focal infections are less common [1,2,3,4]. Occupational contact with pigs or raw pork products constitutes the primary risk factor, particularly among farmers, abattoir workers, and meat handlers in endemic regions of Asia [3,5]. Given its dual importance in swine health and human disease, *S. suis* represents a One Health concern in endemic areas.

In China, serotype 2 strains belonging to sequence type 7 (ST7) have been responsible for two large-scale outbreaks of streptococcal toxic shock-like syndrome (STSLS) in 1998 and 2005, causing high mortality [6,7,8]. These highly virulent ST7 strains are genetically distinct from the globally distributed ST1 lineage and carry a unique repertoire of virulence factors [9,10].

Pyogenic spondylitis (vertebral osteomyelitis) with epidural abscess represents an exceptionally rare manifestation of *S. suis* infection. Only a limited number of cases of *S. suis* spondylodiscitis have been reported worldwide [11,12,13,14]. Diagnosis is frequently delayed because initial symptoms mimic degenerative spine disease, as occurred in our patient. Tissue mNGS has emerged as a valuable culture-independent diagnostic tool for identifying pathogens in deep-seated infections, particularly when prior antimicrobial therapy may compromise conventional culture yield [15,16].

Here, we describe a farmer with serotype 2 ST7 *S. suis* infection presenting as pyogenic spondylitis with epidural abscess, confirmed by tissue mNGS. We integrate clinical, microbiological, antimicrobial susceptibility, and whole-genome sequencing data to characterize this case and discuss implications for diagnosis and prevention.

## 2. Materials and Methods

### 2.1. Clinical Microbiology and Tissue mNGS

Peripheral venous blood was collected prior to initiation of antimicrobial therapy and inoculated at the bedside into aerobic and anaerobic blood culture bottles. Bottles were incubated at 37 °C in an automated blood culture system (Becton, Dickinson and Company, Franklin Lakes, NJ, USA) until flagged positive. Positive bottles underwent Gram staining and were subcultured onto 5% sheep blood agar (35 ± 2 °C, 5% CO_2_, 18–24 h) to obtain pure colonies. Well-isolated colonies were identified by MALDI-TOF MS (bioMérieux, Marcy l’Étoile, France) with on-plate formic acid extraction per the manufacturer’s instructions [17]. Identification was considered reliable when the system reported a “good identification” defined as a single identification choice with ≥60% confidence. Results reported as “low discrimination” or “no identification” were considered inconclusive.

Lumbar biopsy from the L2–L5 region was submitted to a commercial clinical laboratory (Dian Diagnostics, Hangzhou, China) for mNGS using a validated clinical workflow as previously described [15,18]. Briefly, nucleic acids were extracted, and DNA libraries were prepared and sequenced on an Illumina NextSeq platform (Illumina, Inc., San Diego, CA, USA). Reads were aligned to a comprehensive microbial reference database, and pathogen identification was based on species-specific read counts exceeding predefined thresholds.

### 2.2. Antimicrobial Susceptibility Testing (AST)

Minimal inhibitory concentrations (MICs) were determined by broth microdilution. MICs were read using an Vizion automated plate reader (Trek Diagnostic Systems Ltd., East Grinstead, UK).

Results were interpreted according to CLSI M100 (2022) [19] and EUCAST (v14.0, 2024) [20]. The antimicrobial panel included penicillin, amoxicillin, cefotaxime, cefepime, meropenem, vancomycin, teicoplanin, chloramphenicol, erythromycin, clindamycin, tetracycline, levofloxacin, moxifloxacin, linezolid, and trimethoprim-sulfamethoxazole. As no species-specific breakpoints are available for *S. suis*, MICs were interpreted using CLSI M100/EUCAST viridans group streptococci surrogate criteria where surrogate breakpoints were available.

### 2.3. Whole-Genome Sequencing and Bioinformatics

Genomic DNA was extracted from overnight blood agar cultures using a commercial DNA extraction kit according to the manufacturer’s protocol. DNA quality and quantity were assessed prior to library preparation. Paired-end libraries were prepared and sequenced on an Illumina NextSeq platform.

Raw sequencing data were quality-controlled using fastp (v0.23.1) [21]. Taxonomic classification was performed using Kraken2 (v2.1.2) to confirm species identity [22]. De novo assembly was conducted using SPAdes (v3.15.5) [23]. Genome annotation was performed using Prokka (v1.14.6) [24].

Multilocus sequence typing (MLST) was assigned using the *S. suis* MLST scheme hosted at PubMLST (https://pubmlst.org, accessed on 11 November 2025) [25]. In silico serotyping was performed by analysis of the capsular polysaccharide (cps) locus as previously described [26].

Acquired antimicrobial resistance genes were identified using ResFinder 4.7.2 with default thresholds (identity ≥ 90%, coverage ≥ 80%) [27]. Virulence-associated genes were screened using MyDbFinder 2.0 against a custom *S. suis* virulence gene database compiled from published literature [28,29], applying identity ≥ 90% and coverage ≥ 80% thresholds. Putative zoonotic virulence factors (PZVFs) were screened using tblastn against the *S. suis* PZVFs from Roodsant et al. [30], applying identity ≥ 85% and coverage ≥ 80% thresholds.

### 2.4. Phylogenetic Reconstruction and cgSNP Analysis

For pan-genome phylogenetic analysis, 65 representative public genomes and the study isolate SS-JX2025-01 were selected. All genomes were annotated using Prokka v1.14.6 [24], and the pan-genome was constructed with Roary v3.13.0 [31]. Recombinant regions were removed from the core gene alignment using Gubbins v2.4.1 [32]. A maximum likelihood phylogeny was inferred with IQ-TREE v2.4.0 using ModelFinder (-m MFP), with node support assessed by 1000 ultrafast bootstraps (-B 1000) and 1000 SH-aLRT tests (-alrt 1000) [33]. The final tree was visualized and annotated with metadata using the ggtree v3.8.0 package in R [34].

For core genome SNP (cgSNP) analysis of the ST7 clonal complex, 19 public genomes and SS-JX2025-01 were analyzed. SNPs were called against reference *S. suis* strain 05ZYH33 (RefSeq Assembly GCF_000014305.1) using Snippy v4.6.0 (https://github.com/tseemann/snippy, accessed on 11 November 2025). Following recombination filtering with Gubbins v2.4.1 [32], a maximum likelihood tree was constructed using IQ-TREE v2.4.0 as described above. Pairwise SNP distances were calculated using snp-dists v0.8.2 (https://github.com/tseemann/snp-dists, accessed on 28 June 2024) to quantify genetic relatedness.

Bioinformatic technical support for phylogenetic analyses was provided by Hangzhou Adicon Clinical Laboratories Co., Ltd. (Hangzhou, China).

## 3. Results

### 3.1. Case Presentation

A 65-year-old male farmer from Daqiao Town, Nanhu District, Jiaxing (Zhejiang, China) presented with fever (up to 38.9 °C) and low back pain after accidentally falling into a soft-shelled turtle pond on 21 June 2025. He had a history of long-term bare-handed handling of raw pig lungs (purchased from a local market) to feed the turtles, reporting frequent skin breaks due to the lack of protective gloves and timely disinfection.

On 23 June, lumbar radiographs showed degenerative changes only, and conservative management was advised. On 25 June, he developed recurrent fever with chills, urinary incontinence, fatigue, poor appetite, and slowed responses, prompting admission to the intensive care unit (ICU) for “infectious fever of unknown origin.” He also reported subjective hearing decline.

Initial laboratory investigations revealed marked inflammatory response: C-reactive protein (CRP) 269.10 mg/L, white blood cell (WBC) count 18.69 × 10^9^/L (neutrophils 89.5%), procalcitonin (PCT) 3.460 ng/mL, blood glucose 15.40 mmol/L, sodium 133.0 mmol/L, arterial pH 7.48, and pCO_2_ 31.70 mmHg. Chest computed tomography (CT) showed left lower lobe bronchial obstruction with distal atelectasis; head CT revealed no acute abnormalities. Empiric antimicrobial therapy with cefoperazone-sulbactam plus moxifloxacin was initiated.

On 27 June, blood cultures flagged positive, and *S. suis* was identified by MALDI-TOF MS. Antimicrobial therapy was continued; body temperature normalized by 1 July.

On 9 July, fever recurred with worsening lumbar pain. Contrast-enhanced MRI revealed multilevel thoracolumbar infection involving T12–L5, with sub-ligamentous beneath the posterior longitudinal ligament (PLL) and an epidural abscess spanning L2–L5, L4–L5 spinal canal stenosis, and paravertebral soft-tissue edema. Lumbar puncture and CT-guided biopsy were performed. Tissue mNGS (Dian Diagnostics, Hangzhou, China) detected *S. suis* sequences, providing concordant evidence supporting pathogen involvement at the vertebral focus.

Clinical status improved with continued targeted antimicrobial therapy; hearing partially improved. The patient was discharged on 24 July on oral antimicrobial therapy. At follow-up, he remained afebrile, with persistent back pain limiting long-distance ambulation (short-distance independent walking preserved) and mild residual hearing loss.

Exposure and household context: The patient kept pigs, poultry, and a dog at his household. A pigsty was managed primarily by his wife, and a turtle pond was located adjacent to the home. He denied contact with sick or dead pigs. His wife remained asymptomatic throughout.

### 3.2. Microbiology and AST

Blood culture yielded a pure growth of α-hemolytic streptococci; the isolate was designated SS-JX2025-01. MALDI-TOF MS identification was *S. suis* with high confidence. Tissue mNGS from the lumbar biopsy independently detected *S. suis*, consistent with the blood culture identification and supporting pathogen involvement at the vertebral focus.

Antimicrobial susceptibility testing demonstrated that the isolate was susceptible to all tested β-lactams (penicillin MIC ≤ 0.03 mg/L, amoxicillin ≤ 0.25 mg/L, cefotaxime ≤ 0.5 mg/L, cefepime ≤ 0.5 mg/L, meropenem ≤ 0.06 mg/L), glycopeptides (vancomycin ≤ 0.5 mg/L, teicoplanin ≤ 0.25 mg/L), chloramphenicol (≤2 mg/L), levofloxacin (≤0.5 mg/L), and linezolid (≤1 mg/L). Resistance was observed to erythromycin (>2 mg/L), clindamycin (>1 mg/L), and tetracycline (>8 mg/L). For moxifloxacin (≤0.25 mg/L) and trimethoprim-sulfamethoxazole (≤0.5 mg/L), MICs were reported without categorical interpretation as no validated *S. suis* or surrogate breakpoints were available.

### 3.3. Genome Assembly and General Features

Kraken2 analysis confirmed the species identity as *S. suis*, with no evidence of significant contamination. The average sequencing coverage was >500×. The draft genome assembly comprised 33 contigs with an N50 of 76520 bp, a total size of 1.96 Mb, and a GC content of 41.13%, consistent with typical *S. suis* genomes.

In silico analysis assigned the isolate to serotype 2 and sequence type 7 (ST7).

### 3.4. Antimicrobial Resistance Genes

ResFinder analysis identified four acquired resistance genes: *erm*(B) conferring macrolide-lincosamide-streptogramin B resistance, *tet*(O) and *tet*(40) mediating tetracycline resistance via ribosomal protection, and ant(6)-Ia encoding aminoglycoside resistance (streptomycin class). All genes were detected with high sequence identity and coverage, meeting the predefined thresholds. These genotypic findings were concordant with the observed phenotypic resistance to erythromycin, clindamycin, and tetracycline. Notably, the presence of *ant*(6)-Ia predicts resistance to streptomycin, which was not included in our phenotypic panel.

### 3.5. Virulence Factor Profile

MyDbFinder analysis identified 86 of 99 queried virulence-associated genes. The complete gene list is provided in Supplementary Table S1.

**Figure 1:**
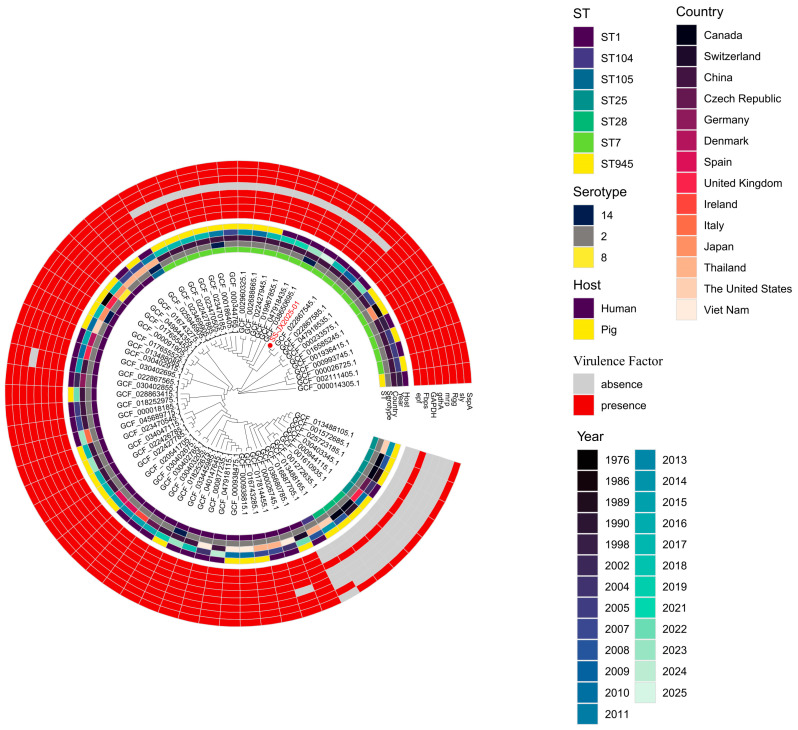
Maximum-likelihood phylogenetic tree of *Streptococcus suis* based on core-genome alignment. The study isolated SS-JX2025-01 (highlighted in red) clusters within the Sequence Type 7 (ST7) lineage. Concentric rings represent metadata from inner to outer: (1) Sequence Type (ST); (2) Serotype; (3) Country of origin; (4) Year of isolation; and (5) Host origin. The outermost heatmap illustrates the presence or absence of eight key virulence-associated genes (*epf*, *Fbps*, *GAPDH*, *gdhA*, *mrp*, *Rgg*, *sly*, and *SspA*).

Among the eight key virulence markers highlighted in the phylogenetic analysis (Figure 1), the isolate was positive for seven: *epf* (extracellular protein factor), *Fbps* (fibronectin-binding protein), *GAPDH* (glyceraldehyde-3-phosphate dehydrogenase), *gdhA* (glutamate dehydrogenase), *Rgg* (transcriptional regulator), *sly* (suilysin), and *SspA* (subtilisin-like protease). Only *mrp* (muramidase-released protein) was absent, yielding an *epf*^+^/*sly*^+^/*mrp*^−^ profile.

Other notable virulence determinants included adhesins *sao*, *lmb*, and *sadP*/*fhbp*, immune-evasion enzymes *zmpC*, *IdeS*, and *Hyl*, and sortase-assembled pilus loci *srtBCD* and *srtF* pilus, whereas *fhb*, *IgdE*, and *srtG* pilus were absent. Importantly, the 89K pathogenicity island (PAI) markers (*salK*/*salR*, *VirB4*, *VirD4*/*TraG*), which have been proposed as key contributors to the hypervirulent phenotype in the 2005 Sichuan outbreak strains [9], were not detected.

Additionally, we screened SS-JX2025-01 for the 26 putative zoonotic virulence factors (PZVFs) defined by Roodsant et al. [30]. Overall, 20 of 26 PZVFs were detected: cbp40/omp40, cps2B, cps2E, cps2F, cps2G, cps2J, cps2L, Fhb_2, hylA, IdeS, NeuB, PnuC, rfeA, RggTR, Sbp1, Sbp2, sly, SP1, Tran, and Zmp. Six PZVFs were not detected: Fhb_1, Hhly3, IgdE, MRP, NisK, and NisR.

### 3.6. Phylogenetic Analysis

The core-genome phylogenetic analysis (pan-genome), based on a high-resolution alignment generated by Roary, placed SS-JX2025-01 within a well-supported clade comprising ST7 isolates (Figure 1). This clade was predominantly composed of strains from China, clearly separating from the globally distributed ST1 lineage. Within the ST7 clade, three subclades were resolved. Further cgSNP analysis identified GCF_022867545.1 (human blood isolate; Fujian, China; 2017) as the closest genome to SS-JX2025-01, differing by only 7 cgSNPs; both shared an *epf*^+^/*sly*^+^/*mrp*^−^ profile. In contrast, SS-JX2025-01 differed by 33 cgSNPs from the 2005 Sichuan outbreak genome GCF_000026725.1, which carried an *epf*^+^/sly^+^/mrp^+^ profile. These findings confirm that SS-JX2025-01 belongs to the virulent Chinese ST7 lineage while demonstrating lineage-dependent variation in the *mrp* gene.

## 4. Discussion

This case illustrates pyogenic spondylitis with epidural abscess as a rare but severe manifestation of *S. suis* serotype 2 ST7 infection. While *S. suis* classically presents as meningitis or sepsis [1,2], vertebral osteomyelitis is uncommon and may be misdiagnosed as degenerative spine disease, as initially occurred in this patient. The diagnosis was established only after MRI revealed characteristic findings and tissue mNGS detected *S. suis* in the vertebral biopsy specimen, providing concordant evidence supporting a deep spinal focus.

*S. suis*-associated spondylodiscitis (pyogenic spondylitis/vertebral osteomyelitis) has been reported sporadically worldwide but remains an uncommon manifestation of invasive *S. suis* disease. In a recent systematic review, Romay-Lema et al. [11] summarized 17 published cases reported between 1994 and 2020, showing that most patients were middle-aged men (76.5%; mean age 57.6 years), frequently with exposure to pigs or pork (70.6%) and predominant lumbar involvement (70.6%); concomitant meningitis was common (70.6%), and β-lactams were used with a mean treatment duration of 53.2 days, with one recurrence and no deaths reported. Notably, diagnosis was most often established by blood and/or cerebrospinal fluid cultures, whereas vertebral/disc culture confirmation was rare (reported in only one case), highlighting the diagnostic challenges of deep spinal infection. Since that review, additional reports have continued to document this entity, including a cluster of human infections in Jeju, South Korea, in which one patient developed infectious spondylitis (lumbar L2–L3) and *S. suis* was isolated from blood, with strain-relatedness assessed by whole-genome sequencing [35]. A recent case report from Portugal further emphasized the clinical complexity of multisegmental spondylodiscitis and the difficulty of obtaining microbiological confirmation from CT-guided spinal biopsies [12]. Our case shares typical features with prior reports (clear exposure history and predominant lumbar involvement) but is notable for extensive epidural/sub-PLL abscess without a diagnosis of concomitant meningitis; etiological confirmation was supported by lumbar biopsy-based mNGS together with WGS characterization of the bloodstream isolate, illustrating a potential role for metagenomic approaches in deep-seated infections, particularly when sampling occurs after antibiotic exposure.

The application of tissue mNGS served a useful adjunct in this case. Although blood cultures had identified *S. suis* earlier, the tissue mNGS result provided concordant evidence supporting the vertebral focus as an active site of infection rather than merely a consequence of transient bacteremia, as the mNGS-derived *S. suis* reads mapped to multiple distinct contigs of the blood isolate WGS assembly with high nucleotide identity. Notably, mNGS was able to detect the pathogen despite the biopsy being obtained after initiation of antimicrobial therapy, further underscoring its utility when conventional culture sensitivity may be compromised. This finding has therapeutic implications, as vertebral osteomyelitis requires prolonged antimicrobial therapy (typically 6–12 weeks) compared with uncomplicated bacteremia [36]. Tissue mNGS is increasingly recognized as a valuable adjunct for diagnosing deep-seated, culture-negative, or polymicrobial infections, particularly when prior antimicrobial exposure may reduce culture sensitivity [15,16].

Genomic characterization confirmed SS-JX2025-01 as serotype 2, ST7, a lineage responsible for two large-scale outbreaks of STSLS in Jiangsu (1998) and Sichuan (2005) provinces, with case-fatality rates exceeding 20% [6,7,8]. Our phylogenetic analysis demonstrates that SS-JX2025-01 clusters within this distinct Chinese ST7 clade, which is genetically separated from the globally predominant ST1 lineage (Figure 1). The ST7 clade’s mixed human-porcine composition underscores its continued zoonotic transmission potential.

Interestingly, SS-JX2025-01 lacked the mrp gene, traditionally considered part of the “classic virulent” profile (*mrp*^+^/*epf*^+^/*sly*^+^). This virulence-marker combination was originally derived from phenotypic studies in European serotype 2 strains, in which MRP, EF (EPF), and suilysin (SLY) were associated with virulent clinical backgrounds [37,38]. However, this profile is not universal among all virulent strains. In our dataset, several ST7 genomes exhibited an *epf*^+^/*sly*^+^/*mrp*^−^ profile (e.g., SS-JX2025-01 and its closest relative GCF_022867545.1), and large-scale genomic analyses have similarly documented *mrp*-negative strains among invasive human isolates across multiple lineages [10]. The independent loss of *mrp* is plausible given that *mrp*, *epf*, and *sly* reside at distinct chromosomal loci in the *S. suis* genome rather than being co-located within a single pathogenicity island (e.g., separated by >1 Mb in strain SC84). Despite lacking *mrp*, SS-JX2025-01 retained epf and sly alongside numerous other virulence determinants, including *SspA*, *Rgg*, *gdhA*, *Fbps*, *sao*, *lmb*, *sadP*/*fhbp*, *zmpC*, and *IdeS*. Experimental and epidemiological evidence suggests that MRP is better characterized as a virulence marker than an essential virulence factor [28]; the severe invasive disease in our patient despite mrp absence is consistent with this view.

Notably, SS-JX2025-01 lacked the canonical 89K pathogenicity island (89K PAI) markers (*salK*/*salR*, *VirB4*, *VirD4*/*TraG*). The 89K PAI was initially proposed as a key determinant of the hypervirulent phenotype in the 2005 Sichuan outbreak strains [9]. However, subsequent surveillance in southern China revealed that 9 of 10 clinical *S. suis* strains isolated during 2008–2015 had lost the 89K PAI entirely or partially, yet still caused severe infections including meningitis, indicating that the 89K PAI is not an essential element for *S. suis* virulence [39]. Our case further supports this observation.

The antimicrobial resistance profile of SS-JX2025-01, susceptibility to β-lactams but resistance to macrolides, lincosamides, and tetracyclines, is consistent with patterns widely reported among *S. suis* isolates from pigs in China, where resistance rates to tetracyclines and macrolides exceed 95% while β-lactam susceptibility remains high [40,41]. The *erm*(B) and *tet*(O)/*tet*(40) genes detected in our isolate are the predominant resistance determinants in Chinese *S. suis* populations, commonly carried on integrative and conjugative elements (ICEs) and likely reflecting selective pressure from antimicrobial use in swine husbandry [40,41]. Fortunately, first-line β-lactam agents remain effective, permitting successful treatment in this case. However, the high prevalence of macrolide resistance has implications for empiric therapy in penicillin-allergic patients, for whom alternative agents must be carefully selected based on susceptibility testing.

The epidemiological investigation highlighted classical risk factors for *S. suis* infection. The patient’s long-term occupation as a farmer with routine bare-handed handling of raw pig organs, combined with frequent skin breaks, provided a direct portal of entry. The soft-shelled turtle pond, while not a direct source of *S. suis*, may have contributed to skin maceration or minor injuries that facilitated pathogen entry. Importantly, his wife, who managed the pigsty but did not handle raw pork, remained uninfected, emphasizing that direct contact with contaminated tissues is the key exposure [3,5].

The mechanism of vertebral involvement in *S. suis* infection likely involves hematogenous seeding of the highly vascularized vertebral endplates during bacteremia [42]. Similarly to other reported cases of *S. suis* spondylodiscitis, the insidious onset over days to weeks likely contributed to diagnostic delay in our patient [11,12].

This case has several limitations. First, porcine source isolates were not available for comparative genomic analysis, precluding definitive confirmation of the transmission chain. Second, formal audiometric assessment was not performed; hearing loss was based on subjective report. Third, optimal antimicrobial duration for *S. suis* vertebral osteomyelitis remains undefined; we extrapolated from general spondylodiscitis guidelines recommending 6–12 weeks of therapy [36]. Fourth, the etiological diagnosis rests primarily on blood culture with MALDI-TOF MS identification; the tissue mNGS finding provides concordant adjunctive evidence, but because the biopsy was obtained after initiation of antimicrobial therapy, only 10 *S. suis* reads were recovered (mapping to 9 distinct contigs of the WGS assembly with 98–100% identity), precluding a definitive strain-level comparison between the tissue and blood isolates. Finally, this represents a single case and may not be generalizable to all presentations of *S. suis* spondylitis.

From a public health perspective, this case reinforces the importance of preventive measures for individuals with occupational exposure to pigs and pork products: use of protective gloves during handling of raw meat, prompt wound disinfection after any skin injury, and seeking early medical attention for febrile illness following exposure [4]. Enhanced awareness among clinicians in endemic areas is essential, particularly for atypical presentations such as spondylitis that may initially mimic degenerative conditions.

## 5. Conclusions

We report a case of *S. suis* serotype 2 ST7 causing pyogenic spondylitis with epidural abscess in a Chinese farmer, supported by tissue mNGS and characterized by whole-genome sequencing. Phylogenetic analysis placed the isolate within the virulent Chinese ST7 clade, although it lacked the 89K PAI. While spondylodiscitis remains an uncommon manifestation of *S. suis* infection, this case serves as a reminder that the clinical spectrum of invasive *S. suis* disease extends beyond the more typical presentations of meningitis and septicemia. Clinicians in endemic regions should consider *S. suis* in the differential diagnosis of spondylodiscitis in patients with relevant occupational or dietary exposure, particularly when conventional cultures are negative. Tissue mNGS can serve as a useful adjunctive diagnostic tool for confirming deep-seated infections. Continued surveillance and preventive measures targeting high-risk occupational groups remain warranted.

## Data Availability

The raw data supporting the conclusions of this article are not publicly available because they are part of an ongoing larger research project but will be provided by the corresponding author on reasonable request.

## Supplementary Materials

The following supporting information can be downloaded at: https://www.mdpi.com/article/10.3390/pathogens15030314/s1, Table S1: Complete list of 99 virulence-associated genes screened in *S. suis* strain SS-JX2025-01.

## References

[1] Gottschalk M., Segura M., Xu J. (2007). *Streptococcus suis* infections in humans: The Chinese experience and the situation in North America. Anim. Health Res. Rev..

[2] Huong V.T.L., Ha N., Huy N.T., Horby P., Nghia H.D.T., Thiem V.D., Zhu X., Hoa N.T., Hien T.T., Zamber J. (2014). Epidemiology, clinical manifestations, and outcomes of *Streptococcus suis* infection in humans. Emerg. Infect. Dis..

[3] Wertheim H.F.L., Nghia H.D.T., Taylor W., Schultsz C. (2009). *Streptococcus suis*: An emerging human pathogen. Clin. Infect. Dis..

[4] Lun Z.R., Wang Q.P., Chen X.G., Li A.X., Zhu X.Q. (2007). *Streptococcus suis*: An emerging zoonotic pathogen. Lancet Infect. Dis..

[5] Nghia H.D.T., Tu L.T.P., Wolbers M., Thai C.Q., Hoang N.V.M., Nga T.V.T., Thao L.T.P., Phu N.H., Chau T.T.H., Sinh D.X. (2011). Risk factors of *Streptococcus suis* infection in Vietnam: A case-control study. PLoS ONE.

[6] Yu H., Jing H., Chen Z., Zheng H., Zhu X., Wang H., Wang S., Liu L., Zu R., Luo L. (2006). Human *Streptococcus suis* outbreak, Sichuan, China. Emerg. Infect. Dis..

[7] Tang J., Wang C., Feng Y., Yang W., Song H., Chen Z., Yu H., Pan X., Zhou X., Wang H. (2006). Streptococcal toxic shock syndrome caused by *Streptococcus suis* serotype 2. PLoS Med..

[8] Ye C., Zhu X., Jing H., Du H., Segura M., Zheng H., Kan B., Wang L., Bai X., Zhou Y. (2006). *Streptococcus suis* sequence type 7 outbreak, Sichuan, China. Emerg. Infect. Dis..

[9] Chen C., Tang J., Dong W., Wang C., Feng Y., Wang J., Zheng F., Pan X., Liu D., Li M. (2007). A glimpse of streptococcal toxic shock syndrome from comparative genomics of *S. suis* 2 Chinese isolates. PLoS ONE.

[10] Weinert L.A., Chaudhuri R.R., Wang J., Peters S.E., Corander J., Jombart T., Baig A., Howell K.J., Vehkala M., Välimäki N. (2015). Genomic signatures of human and animal disease in the zoonotic pathogen *Streptococcus suis*. Nat. Commun..

[11] Romay-Lema E.M., Ventura-Valcárcel P., Iñiguez-Vázquez I., García-Pais M.J., García-Garrote F., Rabuñal-Rey R., Alonso M.P., Corredoira-Sánchez J. (2022). *Streptococcus suis* spondylodiscitis: 2 new cases and a literature review. Enferm. Infecc. Microbiol. Clin..

[12] Vieira J.C., Santos M.M., Afonso J.V., de Magalhães M.S., Teotónio A.C. (2024). Infectious Multisegmental Spondylodiscitis in a Swine Farmer: Diagnostic and Therapeutic Insights. Cureus.

[13] Huang Y.T., Teng L.J., Ho S.W., Hsueh P.R. (2005). *Streptococcus suis* infection. J. Microbiol. Immunol. Infect..

[14] Tsai H.C., Lee S.S., Wann S.R., Huang T.S., Chen Y.S., Liu Y.C. (2005). *Streptococcus suis* meningitis with ventriculoperitoneal shunt infection and spondylodiscitis. J. Formos. Med. Assoc..

[15] Gu W., Deng X., Lee M., Sucu Y.D., Abers M., Pohl-Koeppe A., Wang C., Chiu C.Y., Miller S. (2021). Rapid pathogen detection by metagenomic next-generation sequencing of infected body fluids. Nat. Med..

[16] Chiu C.Y., Miller S.A. (2019). Clinical metagenomics. Nat. Rev. Genet..

[17] Patel R. (2015). MALDI-TOF MS for the diagnosis of infectious diseases. Clin. Chem..

[18] Simner P.J., Miller S., Carroll K.C. (2018). Understanding the promises and hurdles of metagenomic next-generation sequencing as a diagnostic tool for infectious diseases. Clin. Infect. Dis..

[19] Clinical and Laboratory Standards Institute (2022). Performance Standards for Antimicrobial Susceptibility Testing.

[20] The European Committee on Antimicrobial Susceptibility Testing (2024). Breakpoint Tables for Interpretation of MICs and Zone Diameters, Version 14.0. https://www.eucast.org/clinical_breakpoints.

[21] Chen S., Zhou Y., Chen Y., Gu J. (2018). fastp: An ultra-fast all-in-one FASTQ preprocessor. Bioinformatics.

[22] Wood D.E., Lu J., Langmead B. (2019). Improved metagenomic analysis with Kraken 2. Genome Biol..

[23] Bankevich A., Nurk S., Antipov D., Gurevich A.A., Dvorkin M., Kulikov A.S., Lesin V.M., Nikolenko S.I., Pham S., Prjibelski A.D. (2012). SPAdes: A new genome assembly algorithm and its applications to single-cell sequencing. J. Comput. Biol..

[24] Seemann T. (2014). Prokka: Rapid prokaryotic genome annotation. Bioinformatics.

[25] Jolley K.A., Bray J.E., Maiden M.C.J. (2018). Open-access bacterial population genomics: BIGSdb software, the PubMLST.org website and their applications. Wellcome Open Res..

[26] Okura M., Osaki M., Nomoto R., Arai S., Osawa R., Sekizaki T., Takamatsu D. (2016). Current taxonomical situation of *Streptococcus suis*. Pathogens.

[27] Bortolaia V., Kaas R.S., Ruppe E., Roberts M.C., Schwarz S., Cattoir V., Philippon A., Allesoe R.L., Rebelo A.R., Florensa A.F. (2020). ResFinder 4.0 for predictions of phenotypes from genotypes. J. Antimicrob. Chemother..

[28] Fittipaldi N., Segura M., Grenier D., Gottschalk M. (2012). Virulence factors involved in the pathogenesis of the infection caused by the swine pathogen and zoonotic agent *Streptococcus suis*. Future Microbiol..

[29] Feng Y., Zhang H., Wu Z., Wang S., Cao M., Hu D., Wang C. (2014). *Streptococcus suis* infection: An emerging/reemerging challenge of bacterial infectious diseases?. Virulence.

[30] Roodsant T.J., van der Putten B.C.L., Tamminga S.M., Schultsz C., van der Ark K.C.H. (2021). Identification of *Streptococcus suis* putative zoonotic virulence factors: A systematic review and genomic meta-analysis. Virulence.

[31] Page A.J., Cummins C.A., Hunt M., Wong V.K., Reuter S., Holden M.T., Fookes M., Falush D., Keane J.A., Parkhill J. (2015). Roary: Rapid large-scale prokaryote pan genome analysis. Bioinformatics.

[32] Croucher N.J., Page A.J., Connor T.R., Delaney A.J., Keane J.A., Bentley S.D., Parkhill J., Harris S.R. (2015). Rapid phylogenetic analysis of large samples of recombinant bacterial whole genome sequences using Gubbins. Nucleic Acids Res..

[33] Minh B.Q., Schmidt H.A., Chernomor O., Schrempf D., Woodhams M.D., von Haeseler A., Lanfear R. (2020). IQ-TREE 2: New models and efficient methods for phylogenetic inference in the genomic era. Mol. Biol. Evol..

[34] Yu G., Smith D.K., Zhu H., Guan Y., Lam T.T.Y. (2017). ggtree: An R package for visualization and annotation of phylogenetic trees with their covariates and other associated data. Methods Ecol. Evol..

[35] Kim E.T., Heo S.T., Yoo J.R., Kim M., Kim T.H., Kim Y.R. (2024). Case Report: *Streptococcus suis* Human Infections among Pork Consumers in Jeju, South Korea: Zoonotic Threats. Am. J. Trop. Med. Hyg..

[36] Berbari E.F., Kanj S.S., Kowalski T.J., Darouiche R.O., Widmer A.F., Schmitt S.K., Hendershot E.F., Holtom P.D., Huddleston P.M., Petermann G.W. (2015). 2015 Infectious Diseases Society of America (IDSA) clinical practice guidelines for the diagnosis and treatment of native vertebral osteomyelitis in adults. Clin. Infect. Dis..

[37] Vecht U., Wisselink H.J., Jellema M.L., Smith H.E. (1991). Identification of two proteins associated with virulence of *Streptococcus suis* type 2. Infect. Immun..

[38] Allgaier A., Goethe R., Wisselink H.J., Smith H.E., Valentin-Weigand P. (2001). Relatedness of *Streptococcus suis* isolates of various serotypes and clinical backgrounds as evaluated by macrorestriction analysis and expression of potential virulence traits. J. Clin. Microbiol..

[39] Shi X., Ye H., Wang J., Li Z., Wang J., Chen B., Wen R., Hu Q., Feng Y. (2016). Loss of 89K Pathogenicity Island in Epidemic *Streptococcus suis,* China. Emerg. Infect. Dis..

[40] Li Y., Ma B., Jia X., Wan Y., Ni S., Chen G., Zong X., Jin H., Li J., Tan C. (2025). Population Genomics, Virulence Traits, and Antimicrobial Resistance of *Streptococcus suis* Isolated in China. Microorganisms.

[41] Tan M.F., Liu C.L., Zhou Q.Y., Wei J.Z., Hong J.W., Wan M.C., Zhang F.L., Ji H.Y. (2025). Prevalence and antimicrobial resistance of *Streptococcus suis* isolated from local pig breeds in Jiangxi Province, China. Front. Vet. Sci..

[42] Zimmerli W. (2010). Vertebral osteomyelitis. N. Engl. J. Med..

