# Supplementary material for: Pyogenic Spondylitis with Epidural Abscess Caused by Streptococcus suis Serotype 2 ST7: Tissue mNGS Confirmation and Whole-Genome Characterization of a Human Isolate
Source: Pathogens. 2026 Mar 13;15(3):314. doi: 10.3390/pathogens15030314 (PMC13028986; doi:10.3390/pathogens15030314)
Supplement: Supplementary file 1 [file pathogens-15-00314-s001.zip › pathogens-4145285-supplementary.pdf]

Table S1. Complete list of 99 virulence-associated genes screened in *S. suis* strain SS-JX2025-01.

No.	Virulence-associated genes	
1	*1910HK*	presence
2	*1910HR*	absence
3	*6pgd*	presence
4	*abpb*	presence
5	*AdcR*	presence
6	*lpp103*	presence
7	*apuA*	presence
8	*arcA*	presence
9	*atl*	absence
10	*cbp40/omp40*	presence
11	*CcpA*	presence
12	*cdd*	presence
13	*CiaRH*	presence
14	Collagenase	presence
15	*CovR*	presence
16	*dppIV*	presence
17	*DltA*	presence
18	*Dpr*	presence
19	*endoD*	presence
20	*eno*	presence
21	*epf*	presence
22	*fhb*	absence
23	*sadP/fhbp*	presence
24	*Fbps*	presence
25	*FeoB*	presence
26	*Fur*	presence
27	*gh92*	presence
28	*gdhA*	presence
29	*glnA*	presence
30	*GAPDH*	presence
31	*gpmA*	presence
32	*gtfA*	presence
33	*guaA*	presence
34	*guaB*	presence
35	*Hhly3*	absence
36	*Htps*	presence
37	*HP0245*	presence
38	*HP1717*	presence
39	*Hyl*	presence
40	*IdeS*	presence
41	*zmpC*	presence

42	*IgdE*	absence
43	*ihk*	presence
44	*irr*	presence
45	*Lgt*	presence
46	*lmb*	presence
47	*lspA*	presence
48	*LuxS*	presence
49	*manN*	presence
50	*manL*	presence
51	*mrp*	absence
52	*nadR*	presence
53	*NeuB*	presence
54	*NisK/NisR*	absence
55	*Ofs*	presence
56	*OppA*	presence
57	*SSU05_1548*	presence
58	*SSU05_1549*	presence
59	*PgdA*	presence
60	*PnuC*	presence
61	*prsA*	presence
62	*purA*	presence
63	*purD*	presence
64	putative peptidase	presence
65	*RevS*	presence
66	*rfeA* (RTX toxin)	presence
67	*Rgg*	presence
68	*salK/salR*	absence
69	*scrB*	presence
70	*scrR*	presence
71	*SerS*	presence
72	*SMU-61*-like	presence
73	*sntA*	presence
74	*srtA*	presence
75	*SP1*	presence
76	*SpyM3-0908*	presence
77	*srtBCD*	presence
78	*srtF* pilus	presence
79	*srtG* pilus	absence
80	*ssa*	presence
81	*Ssads*	presence
82	*SsnA* nuclease	presence
83	*SspA*	presence
84	*SsPep*	presence
85	*SSU05_0473*	presence

86	*SSU05_1311*	presence
87	*stp*	presence
88	*sly*	presence
89	*sodA*	presence
90	*sao*	absence
91	*tran*	presence
92	*treR*	presence
93	Trigger factor	presence
94	*TroA*	presence
95	*VirA*	presence
96	*VirB4*	absence
97	*VirD4*	absence
98	*yzpA*	presence
99	*Zur*	absence
